# High-dose Vitamin D_3_ supplementation and glycaemic control in adult males with Type 2 diabetes: a randomised placebo-controlled trial

**DOI:** 10.1080/20523211.2026.2680824

**Published:** 2026-06-05

**Authors:** Muhammed Hassan Nasr, Bassam Abdulrasool Hassan, Noordin Othman, Mahmathi Karuppannan, Shalam Mohamed Hussain, Noorizan Binti Abdulaziz, Mohammed Ahmed Alsarani, Ali Haider Mohammed, Siew Li Teoh, Azhar S. Al-Zubaidi

**Affiliations:** a Clinical Pharmacy Department, Al Rayan National College of Health Sciences, Al-Madinah Al-Munawarah, Saudi Arabia; b Faculty of Pharmacy, Al Rafidain University, Baghdad, Iraq; c Department of Pharmacy Practice, College of Pharmacy, Taibah University, Al-Madinah Al-Munawarah, Saudi Arabia; d Department of Clinical Pharmacy, School of Pharmacy, Management and Science University, University Drive, Off Persiaran Olahraga, Shah Alam, Selangor, Malaysia; e Clinical Pharmacy Department, Faculty of Pharmacy, Universiti Teknologi MARA, Selangor, Malaysia; f Department of Pharmacology, VL College of Pharmacy, Raichur, Karnataka, India; g Laboratory Department, Medical Care Unit, Taibah University, Al-Madinah Al-Munawarah, Saudi Arabia; h School of Pharmacy, Monash University Malaysia, Subang Jaya, Selangor, Malaysia; i College of Medicine, Ashur University, Baghdad, Iraq

**Keywords:** Vitamin D₃, Type 2 diabetes mellitus, insulin resistance, randomised controlled trial, glycaemic control

## Abstract

**Background:**

Vitamin D deficiency is common among individuals with type 2 diabetes mellitus (T2DM) and has been linked to insulin resistance and poor glycaemic control. However, findings from randomised controlled trials remain inconsistent.

**Objective:**

To evaluate the effect of high-dose vitamin D₃ supplementation on insulin resistance and glycaemic parameters in adult males with T2DM.

**Methods:**

This double-blind, randomised, placebo-controlled clinical trial was conducted at Taibah University, Saudi Arabia. Sixty adult males with established T2DM were randomly assigned to receive oral vitamin D₃ (cholecalciferol) 50,000 IU once weekly (n = 30) or a matching placebo (n = 30) for 24 weeks. Fasting blood samples were collected at baseline and post-intervention. The primary outcome was insulin resistance assessed using the Homeostasis Model Assessment of Insulin Resistance (HOMA-IR). Secondary outcomes included fasting blood glucose, plasma insulin, glycated haemoglobin (HbA1c), body mass index, lipid profile, and serum 25-hydroxyvitamin D concentrations. Data were analysed using SPSS version 29 (IBM Corp., Armonk, NY, USA).

**Results:**

Vitamin D₃ supplementation significantly increased serum 25-hydroxyvitamin D concentrations in the intervention group, with no meaningful change in the placebo group. Despite correction of vitamin D deficiency, there were no statistically significant between-group differences at 24 weeks in insulin resistance, fasting blood glucose, plasma insulin, HbA1c, body mass index, or lipid profile. Within-group changes in metabolic parameters were modest and not statistically significant.

**Conclusion:**

In adult males with established T2DM, weekly high-dose vitamin D₃ supplementation for 24 weeks effectively corrected vitamin D deficiency but did not improve insulin resistance or glycaemic control. Vitamin D supplementation should therefore be considered primarily for correcting deficiency rather than for glycaemic management.

## Introduction

Type 2 diabetes mellitus (T2DM) is a major global public health challenge and one of the fastest growing non-communicable diseases worldwide. The International Diabetes Federation estimates that more than 530 million adults are currently living with diabetes, a number projected to exceed 780 million by 2045, with T2DM accounting for approximately 90% of all cases (International Diabetes Federation, 2021). The disease is associated with substantial morbidity, premature mortality, and escalating healthcare costs, largely driven by chronic complications such as cardiovascular disease, nephropathy, neuropathy, and retinopathy (Saeedi et al., 2019; Zheng et al., 2018). Central to the pathophysiology of T2DM is insulin resistance, which precedes overt hyperglycaemia and plays a critical role in disease progression and the development of cardiovascular complications (DeFronzo et al., 2015).

The burden of T2DM is particularly pronounced in the Middle East and North Africa region. Saudi Arabia ranks among the countries with the highest prevalence of diabetes globally, with national estimates indicating that approximately one-quarter of adults are affected, and prevalence rising sharply with age (Al-Rubeaan et al., 2015; Robert et al., 2017). Rapid urbanisation, sedentary lifestyles, obesity, and genetic susceptibility have been identified as major contributors to this epidemic (Alotaibi et al., 2017). Insulin resistance is highly prevalent among Saudi patients with T2DM and represents a major therapeutic challenge, as progressive resistance often necessitates intensification of pharmacotherapy, increasing the risk of adverse effects and healthcare expenditure (Abdul-Ghani & DeFronzo, 2010). Identifying modifiable factors that may improve insulin sensitivity is therefore a clinical priority in this population.

Vitamin D deficiency has emerged as a potential modifiable risk factor linked to insulin resistance and glucose dysregulation. Beyond its classical role in calcium homeostasis and bone metabolism, vitamin D exerts pleiotropic effects through the vitamin D receptor (VDR), which is expressed in pancreatic β-cells, skeletal muscle, adipose tissue, and immune cells (Bouillon et al., 2019; Holick, 2007). Experimental and mechanistic studies suggest that vitamin D may influence insulin secretion, insulin receptor expression, intracellular calcium handling, and inflammatory pathways implicated in insulin resistance (Chiu et al., 2004; Muscogiuri et al., 2014; Pittas et al., 2007). Observational studies have consistently reported inverse associations between serum 25-hydroxyvitamin D levels and insulin resistance, fasting blood glucose, and glycated haemoglobin (HbA1c) (Probosari et al., 2025; Song et al., 2013).

Despite abundant sunshine, vitamin D deficiency is highly prevalent in Saudi Arabia, affecting both the general population and individuals with T2DM (Al-Daghri, 2018; Hu et al., 2023). Cultural practices limiting sun exposure, obesity-related sequestration of vitamin D in adipose tissue, and low dietary intake contribute to this paradox (Wimalawansa, 2018). The coexistence of high rates of vitamin D deficiency and T2DM raises the possibility that correction of vitamin D status could offer metabolic benefits in this setting.

However, evidence from interventional studies remains inconclusive. Several randomised controlled trials and meta-analyses have reported no significant improvement in insulin resistance or glycaemic control following vitamin D supplementation in patients with T2DM, while others have suggested modest benefits, particularly in vitamin D–deficient individuals (Haroon & FitzGerald, 2012; Krul-Poel et al., 2017; Lips et al., 2017; Seida et al., 2014). Heterogeneity in study populations, baseline vitamin D status, dosing regimens, duration of supplementation, and outcome measures has limited the ability to draw definitive conclusions (Chakhtoura et al., 2017). Importantly, well-designed, adequately powered randomised controlled trials from Middle Eastern populations remain scarce.

Recent studies have continued to report inconsistent findings regarding the metabolic effects of vitamin D supplementation in patients with type 2 diabetes and related populations. Emerging evidence from recent clinical and population-based studies suggests that while vitamin D supplementation effectively improves serum vitamin D status, its impact on glycaemic outcomes and metabolic parameters remains variable and may depend on baseline deficiency status, population characteristics, and intervention design (Abdul-Ghani & DeFronzo, 2010; Al-Daghri, 2018; Hu et al., 2023; Lips et al., 2017; Probosari et al., 2025; Wimalawansa, 2018). Against this background, the present double-blind, randomised controlled trial was conducted to evaluate the effect of high-dose vitamin D₃ supplementation on insulin resistance and key glycaemic parameters in adult males with T2DM in Saudi Arabia. Using the homeostasis model assessment of insulin resistance (HOMA-IR) as the primary outcome, and fasting blood glucose, plasma insulin, and HbA1c as secondary outcomes, this study aimed to provide robust clinical evidence to clarify whether vitamin D supplementation confers metabolic benefits in this high-risk population.

## Methods

### Study design and setting

This study was designed as a double-blind, randomised, placebo-controlled clinical trial conducted at the Medical Unit of Taibah University in Al-Madinah Al-Munawarah, Kingdom of Saudi Arabia. The primary objective was to evaluate the effect of vitamin D₃ supplementation on insulin resistance and glycaemic control among adult males with type 2 diabetes mellitus (T2DM). The trial was conducted over a total period of eight months, including a preparatory screening phase from September to November 2017 and an active intervention and follow-up phase from November 2017 to May 2018. The study was conducted in accordance with the principles of the Declaration of Helsinki and followed established methodological standards for the conduct and reporting of randomised controlled trials (World Medical Association, 2013).

### Participants and eligibility criteria

Participants were recruited from outpatient medical clinics affiliated with Taibah University. Male patients with an established diagnosis of T2DM were screened for eligibility. The diagnosis of T2DM was confirmed according to the American Diabetes Association (ADA) diagnostic criteria in force at the time of the study (American Diabetes Association, 2017). Eligible participants were males aged between 18 and 65 years. The study population was restricted to male participants to reduce potential biological and hormonal variability that may influence vitamin D metabolism and glycaemic regulation, particularly variations related to sex hormones and menopausal status. This approach was intended to improve internal consistency and reduce heterogeneity within the study sample.

Patients were excluded if they had received vitamin D supplementation within the preceding six months, had known renal or hepatic disease, or were receiving medications known to interfere with vitamin D metabolism, including systemic corticosteroids, orlistat, cholestyramine, phenytoin, or phenobarbital. Additional exclusion criteria included central obesity defined as a waist circumference greater than 102 cm, previous bariatric or gastric bypass surgery, known thyroid or parathyroid disorders, and current or previous malignancy. These criteria were applied to minimise confounding factors that could independently influence insulin resistance, vitamin D metabolism, or glycaemic outcomes. Information on the duration of diabetes was obtained at baseline based on patient medical records and participant self-report. Information on antidiabetic medications was obtained at baseline from patient medical records. The most commonly used medications included metformin (750 mg twice daily), sitagliptin (50 mg daily), dapagliflozin (10 mg daily), insulin glargine, and insulin aspart. The use of these medications was maintained without changes throughout the 24-week intervention period.

### Sample size determination

The sample size was calculated a priori to ensure adequate statistical power to detect a clinically meaningful difference in insulin resistance between the intervention and control groups. Assuming a two-sided significance level of 5% and a statistical power of 80%, the minimum required sample size was estimated to be 64 participants. Participants were therefore allocated equally between the two study arms, with 32 participants assigned to the intervention group and 32 to the control group.

### Randomisation and allocation concealment

Randomisation was performed using participants’ registration numbers obtained from the Medical Unit’s electronic database. Allocation was carried out by an independent third party from the laboratory department who was not involved in participant recruitment, clinical assessment, data collection, or outcome evaluation. Participants with even registration numbers were assigned to the intervention group, while those with odd registration numbers were allocated to the control group. Allocation concealment was ensured through the use of masked, numbered medication containers identical in appearance for both groups. The randomisation sequence was sealed and stored securely at the Medical Unit and was accessible only in the event of emergency unblinding.

### Blinding

This study employed a double-blind design. Participants, treating physicians, investigators, laboratory personnel, and outcome assessors were all blinded to group allocation throughout the study period. Both the vitamin D₃ capsules and the placebo capsules were identical in size, colour, packaging, and labelling, ensuring maintenance of blinding at all stages of the trial.

### Intervention and control procedures

Participants assigned to the intervention group received oral vitamin D₃ (cholecalciferol) at a dose of 50,000 IU once weekly for a total duration of 24 weeks. The vitamin D₃ preparation used was *Essential Vitamin D₃* (Arnet Pharmaceutical Corp., USA), with each capsule containing 50,000 IU of cholecalciferol. The formulation was free from common allergens and did not contain milk, eggs, wheat, peanuts, fish, shellfish, or derivatives thereof.

Participants in the control group received a placebo vitamin B complex capsule administered orally once weekly for the same duration. The placebo capsules were identical in appearance and packaging to the vitamin D₃ capsules to maintain blinding. The dosing regimen and duration were selected based on established clinical guidance for the correction of vitamin D deficiency and evidence from prior interventional studies demonstrating the biochemical efficacy and safety of high-dose weekly vitamin D supplementation in adults (Heaney et al., 2008; Holick et al., 2011).

### Clinical assessment and follow-up

All participants underwent baseline clinical assessment following enrolment and provision of written informed consent. Demographic data, medical history, comorbidities, and current medications were recorded at baseline only. Anthropometric measurements, including body weight, height, body mass index (BMI), and waist circumference, were obtained using standardised procedures. Blood pressure was measured using a calibrated mercury sphygmomanometer, with two readings taken after a 10-minute rest period and averaged for analysis.

Participants were followed for 24 weeks and reassessed at the end of the intervention period. To enhance adherence and monitor safety, participants were contacted weekly by telephone by the study physician to confirm compliance with the intervention and to identify any adverse events. Vitamin D supplementation was well tolerated, and no serious adverse events were reported during the study period.

### Laboratory measurements

At the end of the run-in period, the active phase of the trial was initiated. Venous blood samples were collected from participants who had provided informed consent for the assessment of fasting blood glucose, glycated haemoglobin (HbA1c), plasma insulin, insulin resistance, and serum 25-hydroxyvitamin D [25(OH)D] concentrations. All venous blood samples were obtained after an overnight fast of at least 12 h at baseline and at the end of the 24-week intervention period. Fasting plasma insulin concentrations were measured using enzyme-linked immunosorbent assay (ELISA), and insulin resistance was subsequently calculated using the Homeostasis Model Assessment of Insulin Resistance (HOMA-IR). Venous blood samples were collected after an overnight fast of at least 12 h at baseline and at the end of the 24-week intervention period. Blood sampling was performed between 10:00 and 12:00 on weekdays by trained laboratory technicians. Samples were centrifuged at 3000 rpm for 30 min at 4°C, and serum was separated and stored at −20°C until analysis.

Serum 25-hydroxyvitamin D [25(OH)D] concentrations were measured using an electrochemiluminescence immunoassay (ECLIA) on the Cobas e411 analyser. Vitamin D deficiency was defined as serum 25(OH)D levels <20 ng/mL, insufficiency as 20–30 ng/mL, and sufficiency as >30 ng/mL (Heaney et al., 2008; Matthews et al., 1985; Wallace et al., 2004). Fasting blood glucose (FBG) and glycated haemoglobin (HbA1c) were measured using photometric assays on the Cobas c311 analyser. Plasma insulin concentrations were determined using enzyme-linked immunosorbent assay (ELISA) on the Cobas e411 analyser.

### Outcome measures

The primary outcome of the study was insulin resistance, assessed using the Homeostasis Model Assessment of Insulin Resistance (HOMA-IR). HOMA-IR was calculated using the standard formula: fasting plasma insulin (µU/mL) multiplied by fasting plasma glucose (mg/dL) divided by 405. A HOMA-IR value ≥2.5 was considered indicative of insulin resistance (Morisky et al., 1986; Pittas et al., 2019).

Secondary outcomes included fasting blood glucose, plasma insulin levels, HbA1c, BMI, lipid profile parameters, and serum 25(OH)D concentrations.

### Adherence assessment

Adherence to the intervention was assessed using two complementary methods. Pill counts were performed at follow-up visits by comparing the number of capsules dispensed with the number returned. In addition, adherence was evaluated using the Morisky–Green–Levine Medication Adherence Scale, with scores categorised according to established criteria (George et al., 2012). Participants with acceptable adherence were included in the final analysis.

### Statistical analysis

Data were recorded on structured investigation report forms and analysed using SPSS version 29 (IBM Corp., Armonk, NY, USA). Continuous variables were summarised as means with standard deviations, while categorical variables were expressed as frequencies and percentages. Between-group comparisons of continuous variables were performed using independent-samples t-tests or Mann–Whitney U tests, as appropriate. Within-group comparisons before and after intervention were conducted using paired t-tests or Wilcoxon signed-rank tests. Categorical variables were compared using chi-square or Fisher’s exact tests. A *p*-value <0.05 was considered statistically significant, and a *p*-value <0.001 was considered highly significant.

### Ethics approval and consent to participate

This study was not prospectively registered in a clinical trial registry. However, the study protocol received formal ethical approval from the Taibah University College of Dentistry Research Ethics Committee (TUCD-REC; approval number TUCD-REC/20171022/Nsr), and all participants provided written informed consent prior to enrolment. The study was conducted in accordance with the Declaration of Helsinki and followed established methodological standards for randomised controlled trials.

## Results

### Participant flow and baseline characteristics

**Figure 1.**
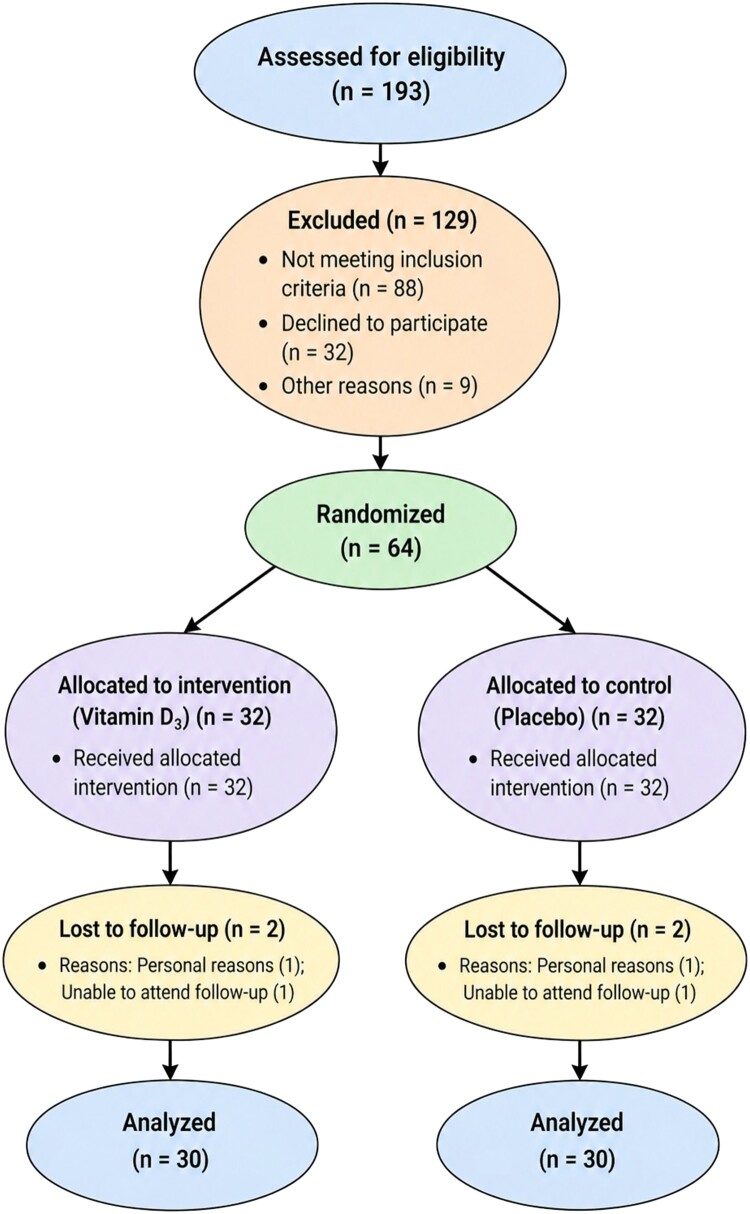
CONSORT flow diagram of participant recruitment, allocation, follow-up, and analysis.

A total of 193 patients were assessed for eligibility, of whom 64 met the inclusion criteria and were randomised to the intervention or control group. During the 24-week follow-up period, four participants were lost to follow-up, resulting in a final analytic sample of 60 participants. A CONSORT flow diagram illustrating participant screening, allocation, follow-up, and analysis is presented in Figure 1. Baseline demographic, metabolic, and biochemical characteristics are presented in Table 1. The mean age of participants was comparable between the intervention and control groups, and the majority of participants in both groups were aged between 41 and 65 years. In addition, the duration of diabetes was comparable between groups, with a mean of 3.7 ± 3.8 years in the intervention group and 3.5 ± 3.4 years in the control group, with no statistically significant difference observed at baseline.

**Table 1.** Baseline demographic, metabolic, and biochemical characteristics of study participants.

Variable	Intervention group (n = 30) Mean ± SD / n (%)	Control group (n = 30) Mean ± SD / n (%)	*p*-value
Age (years), mean ± SD	51 ± 8	51 ± 9	NS
Age 18–40 years, n (%)	4 (13.3%)	5 (16.7%)	NS
Age 41–65 years, n (%)	26 (86.7%)	25 (83.3%)	NS
Duration of diabetes (years), mean ± SD	3.7 ± 3.8	3.5 ± 3.4	NS
Body mass index (kg/m²)	33.39 ± 5.85	31.51 ± 6.61	0.3
HbA1c (%)	8.09 ± 1.82	9.79 ± 1.18	0.04
Insulin resistance (HOMA-IR)	10.11 ± 10.25	9.73 ± 8.18	0.9
Fasting blood glucose (mg/dL)	179.49 ± 77.04	177.06 ± 80.37	0.9
Plasma insulin (µU/mL)	21.13 ± 15.35	21.79 ± 13.76	0.9
Total cholesterol (mg/dL)	194.51 ± 40.56	183.68 ± 42.91	0.3
LDL cholesterol (mg/dL)	120.75 ± 39.90	108.72 ± 38.88	0.2
HDL cholesterol (mg/dL)	37.98 ± 10.16	37.09 ± 9.39	0.7
Triglycerides (mg/dL)	194.06 ± 194.29	191.12 ± 163.81	0.9
Serum 25(OH)D (ng/mL)	14.55 ± 5.70	20.66 ± 7.83	0.001
			

Values are expressed as mean ± standard deviation (SD) or number (%)

There were no statistically significant differences between groups at baseline with respect to body mass index, lipid profile, or insulin resistance. However, baseline HbA1c levels were significantly higher in the control group compared with the intervention group (9.79 ± 1.18 vs 8.09 ± 1.82, *p* = 0.04). Baseline serum 25-hydroxyvitamin D concentrations were lower in the intervention group compared with the control group.

### Glycaemic outcomes

Between-group comparisons of glycaemic outcomes at baseline and after 24 weeks of intervention are shown in Table 2. At baseline, HbA1c, insulin resistance as assessed by HOMA-IR, fasting blood glucose, and plasma insulin levels were similar between the two groups. After 24 weeks, mean HbA1c levels were 7.73 ± 1.56% in the intervention group and 7.15 ± 1.64% in the control group. Mean HOMA-IR values were 8.70 ± 6.57 in the intervention group and 11.03 ± 10.93 in the control group. Fasting blood glucose and plasma insulin concentrations also remained comparable between groups at the end of the study period. No statistically significant between-group differences were observed for any glycaemic parameter after the intervention.

**Table 2.** Effects of vitamin D supplementation on glycaemic outcomes (between-group comparison).

Variable	Intervention Baseline	Intervention 24 weeks	Control Baseline	Control 24 weeks	*p*-value (between groups at 24 weeks)
HbA1c (%)	8.09 ± 1.82	7.73 ± 1.56	9.79 ± 12.18	7.15 ± 1.64	0.2
Insulin resistance (HOMA-IR)	10.11 ± 10.25	8.70 ± 6.57	9.73 ± 8.18	11.03 ± 10.93	0.3
Fasting blood glucose (mg/dL)	179.49 ± 77.04	179.57 ± 74.39	177.06 ± 80.37	172.42 ± 104.27	0.8
Plasma insulin (µU/mL)	21.13 ± 15.35	20.28 ± 13.57	21.79 ± 13.76	24.31 ± 17.87	0.3

Within-group changes in glycaemic parameters over the 24-week period are summarised in Table 3. In the intervention group, insulin resistance, HbA1c, fasting blood glucose, and plasma insulin levels showed numerical reductions following vitamin D supplementation; however, none of these changes reached statistical significance. In the control group, insulin resistance and plasma insulin levels increased numerically over the study period, while fasting blood glucose and HbA1c decreased numerically, with no statistically significant within-group changes observed.

**Table 3.** Within-group changes in glycaemic parameters from baseline to 24 weeks.

Variable	Intervention group (Baseline → 24 weeks)	Δ Mean ± SD	*p*-value	Control group (Baseline → 24 weeks)	Δ Mean ± SD	*p*-value
HbA1c (%)	8.09 → 7.73	−0.36 ± 1.21	0.3	9.79 → 7.15	−2.64 ± 1.92	0.3
Insulin resistance (HOMA-IR)	10.11 → 8.70	−1.41 ± 2.32	0.4	9.73 → 11.03	+1.30 ± 1.62	0.6
Fasting blood glucose (mg/dL)	181.14 → 179.49	−1.65 ± 1.45	0.9	177.06 → 172.42	−4.64 ± 2.51	0.4
Plasma insulin (µU/mL)	21.13 → 20.28	−0.85 ± 1.32	0.6	21.79 → 24.31	+2.52 ± 1.06	0.9

Δ represents the mean change from baseline to 24 weeks.

### Anthropometric and lipid outcomes

Changes in anthropometric and lipid parameters are presented in Table 4. At baseline, body mass index and lipid profile parameters, including total cholesterol, low-density lipoprotein cholesterol, high-density lipoprotein cholesterol, and triglycerides, were similar between the two groups. After 24 weeks, no statistically significant between-group differences were observed in body mass index or lipid parameters.

**Table 4.** Changes in anthropometric and lipid parameters.

Variable	Intervention Baseline	Intervention 24 weeks	Control Baseline	Control 24 weeks	*p*-value
BMI (kg/m²)	33.39 ± 5.85	32.65 ± 5.65	31.51 ± 6.61	31.32 ± 6.93	0.5
HDL cholesterol (mg/dL)	37.98 ± 10.16	35.88 ± 10.20	37.09 ± 9.39	33.22 ± 8.50	0.3
LDL cholesterol (mg/dL)	120.75 ± 39.90	105.27 ± 37.30	108.72 ± 38.88	95.84 ± 34.13	0.3
Triglycerides (mg/dL)	194.06 ± 194.29	202.66 ± 146.97	191.12 ± 163.81	159.80 ± 80.15	0.2
Total cholesterol (mg/dL)	194.51 ± 40.56	181.83 ± 36.43	183.68 ± 42.91	162.55 ± 36.31	0.06

Within the intervention group, body mass index and lipid parameters demonstrated modest numerical changes following supplementation. High-density lipoprotein cholesterol decreased over the study period, while total cholesterol, low-density lipoprotein cholesterol, and triglycerides showed small numerical variations. In the control group, reductions were observed in total cholesterol and triglyceride levels, while body mass index and other lipid parameters remained largely unchanged. Overall, no statistically significant between-group differences were identified for anthropometric or lipid outcomes at the end of the intervention period.

### Vitamin D status

Serum 25-hydroxyvitamin D concentrations before and after the intervention are shown in Table 5. At baseline, mean serum vitamin D levels were 14.55 ± 5.70 ng/mL in the intervention group and 20.66 ± 7.83 ng/mL in the control group, with a statistically significant difference between groups (*p* = 0.001). After 24 weeks of supplementation, mean serum vitamin D concentrations increased significantly in the intervention group to 29.28 ± 12.20 ng/mL, whereas the control group showed a slight decrease to 18.53 ± 5.36 ng/mL. The difference between the two groups after supplementation was statistically significant (*p* = 0.00012).

**Table 5.** Effect of vitamin D supplementation on serum 25-hydroxyvitamin D concentrations.

Group	Baseline 25(OH)D (ng/mL) Mean ± SD	24 weeks 25(OH)D (ng/mL) Mean ± SD	*p*-value
Intervention (n = 30)	14.55 ± 5.70	29.28 ± 12.20	0.00012
Control (n = 30)	20.66 ± 7.83	18.53 ± 5.36	0.09

25(OH)D: 25-hydroxyvitamin D.

Values are expressed as mean ± SD.

At the end of the study, the majority of participants in the intervention group achieved serum vitamin D concentrations approaching sufficiency, whereas vitamin D status remained largely unchanged in the control group.

## Discussion

In this randomised controlled trial, weekly high-dose vitamin D₃ supplementation for 24 weeks resulted in a marked and statistically significant increase in serum 25-hydroxyvitamin D concentrations in the intervention group, confirming the biological efficacy of the supplementation regimen. This finding is consistent with established evidence demonstrating that intermittent high-dose cholecalciferol effectively corrects vitamin D deficiency in adults, including populations with obesity and limited sun exposure (Barzegari et al., 2019; Poolsup et al., 2016). In contrast, serum vitamin D levels in the control group showed no significant improvement, supporting the validity of the blinding and the specificity of the intervention effect.

Despite successful correction of vitamin D deficiency, vitamin D supplementation did not lead to statistically significant improvements in insulin resistance, fasting blood glucose, plasma insulin, or HbA1c when compared with placebo. These results suggest that, in adult males with established type 2 diabetes mellitus, normalisation of vitamin D status alone may be insufficient to produce clinically meaningful improvements in glycaemic control over a 24-week period. This finding aligns with multiple randomised controlled trials reporting neutral effects of vitamin D supplementation on glycaemic indices in patients with type 2 diabetes, even in the presence of substantial increases in serum 25(OH)D concentrations (Autier et al., 2014; Kahn et al., 2014; Manson et al., 2019). Recent evidence further supports these findings. Contemporary studies have reported that vitamin D supplementation, despite effectively correcting deficiency, does not consistently translate into significant improvements in glycaemic control or insulin resistance across different populations. Variability in outcomes has been attributed to differences in study populations, baseline vitamin D status, dosing strategies, and duration of intervention. These findings reinforce the notion that vitamin D may play a supportive rather than a primary therapeutic role in glucose metabolism (Kositsawat et al., 2025; Kositsawat et al., 2026; Mokgalaboni, 2025).

The lack of glycaemic benefit observed in this study is consistent with several large meta-analyses and systematic reviews that have examined the role of vitamin D supplementation in glucose metabolism. While some meta-analyses have reported small reductions in fasting glucose or HOMA-IR, particularly among vitamin D–deficient individuals, the overall effect sizes have generally been modest and of uncertain clinical relevance (Musso et al., 2010; Norman, 2008; Wang et al., 2012). Other high-quality syntheses have concluded that vitamin D supplementation does not significantly improve HbA1c, insulin sensitivity, or diabetes-related outcomes in the general population with type 2 diabetes (Al-Daghri, 2018; Jorde & Grimnes, 2011). Taken together, the present findings support the growing consensus that vitamin D supplementation should not be considered an effective glycaemic control strategy in established type 2 diabetes. Differences in population characteristics may also contribute to discrepancies between the present findings and those reported in the literature. The current study included only male participants, whereas many previous studies have included mixed-sex populations. Sex-specific differences in vitamin D metabolism, hormonal regulation, and insulin sensitivity may influence the metabolic response to supplementation. In addition, the study was conducted in a Middle Eastern population, where genetic background, lifestyle factors, dietary patterns, and sun exposure behaviours differ from those in Western populations. These contextual factors may modify the physiological effects of vitamin D and partly explain the variability in reported outcomes across studies (Hu et al., 2023; Probosari et al., 2025; Robert et al., 2017).

Several factors may explain the neutral metabolic findings observed. First, insulin resistance and hyperglycaemia in type 2 diabetes are multifactorial and strongly influenced by adiposity, duration of disease, pharmacological treatment, physical activity, and dietary intake. Any modest effect of vitamin D on insulin signalling or β-cell function may be overshadowed by these dominant determinants, particularly in patients with longstanding disease (Hu et al., 2023). Second, although vitamin D receptors are expressed in pancreatic β-cells and insulin-sensitive tissues, mechanistic studies suggest that vitamin D may play a permissive or modulatory role rather than acting as a primary regulator of glucose homeostasis (Krul-Poel et al., 2017; Seida et al., 2014). The relatively short mean duration of diabetes (approximately 3–4 years) in this cohort may indicate that many participants were in earlier stages of disease, although the large standard deviations suggest substantial variability. This heterogeneity in disease duration could have influenced responsiveness to vitamin D supplementation, as both early and more established disease states were likely represented (Zheng et al., 2018).

The dosing regimen used in this study may also have influenced the observed outcomes. Although once-weekly high-dose vitamin D₃ supplementation is effective for correcting deficiency, it may result in different pharmacokinetic and physiological responses compared with daily or more frequent dosing regimens. Some evidence suggests that steady daily dosing may provide more consistent serum vitamin D levels and potentially more sustained biological effects. Therefore, it is possible that the once-weekly administration schedule used in the present study may not have provided optimal conditions for detecting subtle metabolic changes in glycaemic parameters (Kositsawat et al., 2026; Manson et al., 2019). However, the significant increase in serum 25-hydroxyvitamin D concentrations observed in the intervention group confirms that the dosing regimen was effective in correcting vitamin D deficiency.

The absence of significant improvements in lipid profile and body mass index further supports the conclusion that vitamin D supplementation did not exert meaningful metabolic effects beyond correcting deficiency. Although observational studies have reported associations between low vitamin D levels and adverse lipid profiles, interventional studies have generally failed to demonstrate consistent lipid-lowering effects, particularly in individuals with type 2 diabetes (Pittas et al., 2007; Song et al., 2013). Nevertheless, some meta-analyses have reported potential beneficial effects of vitamin D supplementation on lipid parameters, particularly reductions in triglyceride levels and, in certain cases, total and LDL cholesterol (MacGirlley et al., 2023). However, these findings remain inconsistent, with several randomised controlled trials and pooled analyses demonstrating no significant effects. Such variability may be explained by differences in baseline vitamin D status, population characteristics, supplementation dose and duration, and study design.

These inconsistencies support the findings of the present study and suggest that the effects of vitamin D on lipid metabolism are likely context-dependent rather than uniform across populations. The modest within-group changes observed in lipid parameters in both study arms are more plausibly attributable to background therapy, lifestyle variation, or regression to the mean rather than a direct effect of vitamin D supplementation. Importantly, this study contributes data from a Middle Eastern population, where vitamin D deficiency and type 2 diabetes are both highly prevalent. Despite abundant sunlight, cultural practices, limited outdoor activity, and obesity contribute to widespread hypovitaminosis D in Saudi Arabia (Lips et al., 2017; Probosari et al., 2025). The present findings suggest that while vitamin D supplementation is effective in correcting deficiency, it should not be expected to improve glycaemic outcomes in isolation. Vitamin D supplementation in this context may therefore be best justified for skeletal health and correction of deficiency rather than as a metabolic intervention.

This study has several strengths, including its randomised, double-blind, placebo-controlled design, standardised laboratory measurements, and assessment of both between-group and within-group changes over a clinically relevant duration. However, certain limitations should be acknowledged. The sample size was modest, which may have limited the power to detect small treatment effects. The study population was restricted to males, which may limit generalizability to females with type 2 diabetes, as sex-specific differences in vitamin D metabolism, hormonal regulation, and insulin sensitivity may influence the response to supplementation. Although antidiabetic medications were maintained throughout the study period, potential variations in individual response or adherence could still have influenced glycaemic outcomes. Additionally, dietary intake and physical activity were not formally quantified, which may have contributed to variability in metabolic parameters. Although assessment of β-cell function using indices such as HOMA-B could provide additional insight into the metabolic effects of vitamin D supplementation, this analysis was not performed in the present study. Future research incorporating β-cell function measures may help further elucidate the potential role of vitamin D in glucose metabolism. Another limitation of this study is the absence of prospective clinical trial registration. Although the study protocol underwent formal ethical review and approval, lack of registration may reduce transparency and limit external verification of predefined outcomes.

An additional limitation is the baseline imbalance in serum vitamin D levels between the intervention and control groups, with higher initial concentrations observed in the control group. This difference may have influenced the study outcomes, as participants in the intervention group had a greater degree of deficiency and may have responded differently to supplementation. Variations in baseline vitamin D status are known to affect responsiveness to supplementation and could potentially confound the interpretation of metabolic effects. The metabolic response to vitamin D supplementation may also be influenced by baseline vitamin D status, with individuals who are more deficient potentially exhibiting greater responsiveness, although this relationship was not formally evaluated in the present study.

In addition, a baseline imbalance in HbA1c levels was observed, with higher initial values in the control group. This difference may have influenced the magnitude of glycaemic changes observed over the study period, as participants with poorer baseline control may exhibit greater reductions due to regression to the mean or intensified clinical attention. Nevertheless, vitamin D supplementation in the intervention group resulted in a substantial increase in serum 25(OH)D concentrations, confirming effective correction of deficiency. Future studies should consider stratification or adjustment based on baseline vitamin D status and glycaemic control to better isolate the true metabolic effects of supplementation.

## Conclusion

In this double-blind, randomised, placebo-controlled trial, weekly high-dose vitamin D₃ supplementation for 24 weeks effectively corrected vitamin D deficiency in adult males with type 2 diabetes mellitus. However, despite the significant increase in serum 25-hydroxyvitamin D concentrations, supplementation did not result in meaningful improvements in insulin resistance, glycaemic control, body mass index, or lipid profile compared with placebo.

These findings suggest that vitamin D₃ supplementation alone is unlikely to provide clinically relevant metabolic benefits in individuals with established type 2 diabetes. In this context, vitamin D supplementation should be considered primarily for the correction of deficiency and maintenance of skeletal health rather than as a strategy for glycaemic management.

## Data Availability

Data and other materials are available upon reasonable request from the corresponding authors.
